# Immune Complex Mediated Glomerulonephritis with Acute Thrombotic Microangiopathy following Newly Detected Hepatitis B Virus Infection in a Kidney Transplant Recipient

**DOI:** 10.1155/2016/3152495

**Published:** 2016-10-09

**Authors:** Tracey Salter, Hannah Burton, Sam Douthwaite, William Newsholme, Catherine Horsfield, Rachel Hilton

**Affiliations:** ^1^Departments of Nephrology and Transplantation, Guy's and St Thomas' NHS Foundation Trust, London, UK; ^2^Department of Virology, Guy's and St Thomas' NHS Foundation Trust, London, UK; ^3^Department of Infectious Diseases, Guy's and St Thomas' NHS Foundation Trust, London, UK; ^4^Department of Histopathology, Guy's and St Thomas' NHS Foundation Trust, London, UK

## Abstract

Hepatitis B virus (HBV) presents a risk to patients and staff in renal units. To minimise viral transmission, there are international and UK guidelines recommending HBV immunisation for patients commencing renal replacement therapy (RRT) and HBV surveillance in kidney transplant recipients. We report the case of a 56-year-old male who was immunised against HBV before starting haemodialysis. He received a deceased donor kidney transplant three years later, at which time there was no evidence of HBV infection. After a further six years he developed an acute kidney injury; allograft biopsy revealed an acute thrombotic microangiopathy (TMA) with glomerulitis, peritubular capillaritis, and C4d staining. Due to a “full house” immunoprofile, tests including virological screening were undertaken, which revealed acute HBV infection. Entecavir treatment resulted in an improvement in viral load and kidney function. HBV genotyping demonstrated a vaccine escape mutant, suggesting “past resolved” infection that reactivated with immunosuppression, though posttransplant acquisition cannot be excluded. This is the first reported case of acute HBV infection associated with immune complex mediated glomerulonephritis and TMA. Furthermore, it highlights the importance of HBV surveillance in kidney transplant recipients, which although addressed by UK guidelines is not currently practiced in all UK units.

## 1. Introduction 

Blood borne virus (BBV) infection presents a risk to both patients and staff in renal centres, with HBV historically associated with outbreaks in haemodialysis units. Globally there exist numerous clinical practice guidelines (CPG) which aim to minimise viral transmission. International CPG recommend that patients who require RRT should be immunised against HBV [1, 2]. There is specific guidance for HBV surveillance in patients receiving regular hospital haemodialysis; those deemed to have achieved protective immunity (anti-hepatitis B surface antibody [anti-HBs] >10 mIU/mL) need to only be tested for hepatitis B surface antigen (HBsAg) annually [1, 2]. In addition, there is international [3] and UK [4] guidance addressing HBV surveillance in kidney transplant recipients.

TMA is characterised by microangiopathic haemolytic anaemia, thrombocytopenia associated with hyaline thrombi, and varying degrees of end organ failure. Numerous and varied causes are recognised, including viral infection [5]. However, there are no reports of TMA in association with acute HBV infection. Here we describe the case of a kidney transplant recipient who developed TMA synchronous to newly detected HBV infection in the absence of other likely causes.

## 2. Case Report

A fifty-six-year-old Arab male developed end stage kidney disease (ESKD) in association with hypertension and type 2 diabetes mellitus and commenced unit haemodialysis. At the time his serology tested negative for HBsAg and HBV core antibody (cAb). In accordance with UK guidance he received a full course of vaccination against HBV infection, receiving HBVaxPro 10 *μ*g at 0, 1, and 6 months; subsequent anti-HBs titre following completion of immunisation was 965 mIU/mL. He was therefore deemed to have acquired protective immunity and was scheduled for annual monitoring of anti-HBs levels, with a booster indicated for any level <100 mIU/mL. One year after immunisation the anti-HBs level was 155 mIU/mL, suggesting that protective immunity had been maintained and that no booster was required.

**Figure 1:**
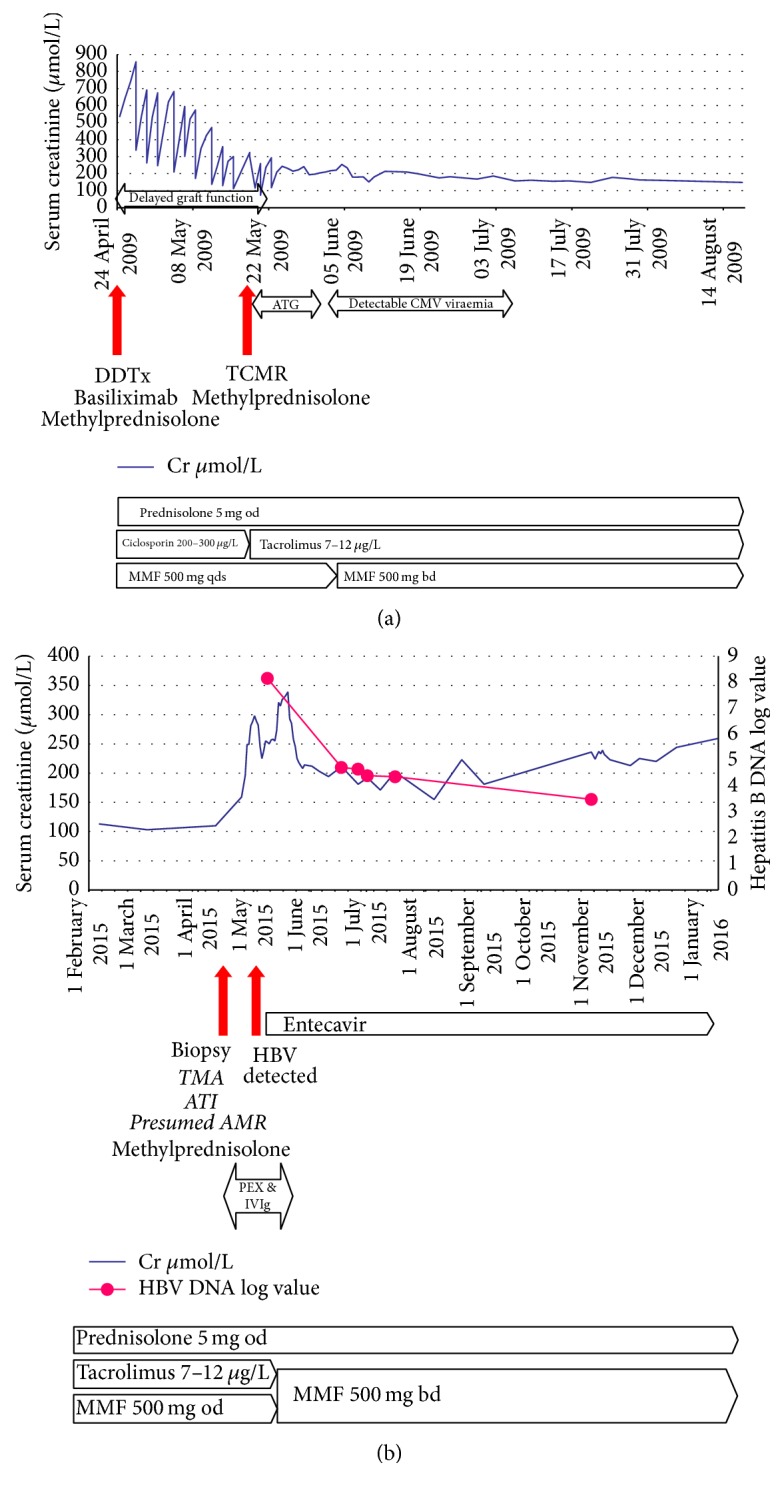
(a) Events following transplantation. (b) Events following his presentation with AKI.

Three years after starting dialysis he received a donation after cardiac death (DCD) deceased donor kidney transplant. At the time he remained HBsAg and cAb negative. Induction immunosuppression comprised basiliximab and methylprednisolone. Maintenance immunosuppression was ciclosporin (target level 200–300 *µ*g/L), mycophenolate mofetil 500 mg four times daily, and corticosteroids. The postoperative course was complicated by delayed graft function and T cell-mediated rejection requiring treatment with pulsed methylprednisolone and antithymocyte globulin (ATG) (Figure 1(a)). Cytomegalovirus (CMV) viraemia was detected and treated with valganciclovir. Thereafter his kidney function improved to a baseline serum creatinine of 130 *μ*mol/L. Maintenance immunosuppression comprised twice-daily tacrolimus with levels maintained between 7 and 12 *µ*g/L, mycophenolate mofetil 500 mg twice daily, and prednisolone 5 mg daily.

Over the next five years his allograft function remained stable. Two immunosuppression-related issues arose:* Escherichia coli* sepsis secondary to cellulitis and a pyogenic granuloma of his thumb. In line with current practice at our renal unit, anti-HBs levels were not checked and no boosters were administered after transplant.

Six years after transplantation he presented to his local hospital nonspecifically unwell, and was found to have acute kidney injury (AKI) with a rise in creatinine from a baseline of 100 *μ*mol/L to almost 350 *μ*mol/L. He was transferred to this hospital for further management (Figure 1(b)).

**Figure 2:**
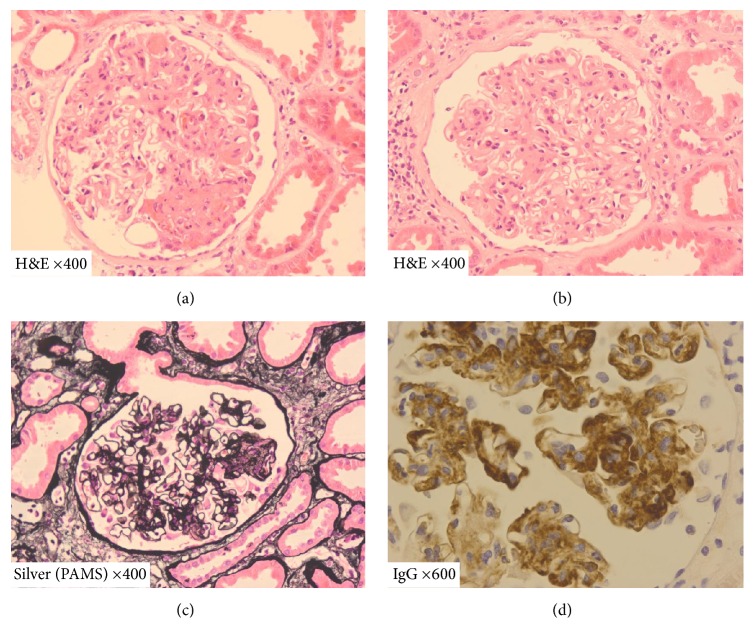
(a) Acute thrombotic occlusion of capillary loops, with segmental endocapillary hypercellularity due to inflammation, with endothelial cell swelling, and with fragmented red blood cells. (b) Mild increase in mesangial cells and moderate expansion of the mesangial matrix. Occasional inflammatory cells in capillary loops. (c) Segmental endocapillary hypercellularity with endothelial cell swelling due to inflammatory cells. (d) Positive staining for immunoglobulin G in the matrix and along glomerular capillary walls. Staining for immunoglobulins A and M and C3 and C1q was also positive.

Allograft biopsy revealed TMA with glomerulitis (g3), mild peritubular capillaritis (ptc1), and acute tubular injury, together with C4d staining (C4d2) (Figure 2). This was attributed to antibody-mediated rejection, although no donor-specific antibody was detected in peripheral blood, and he commenced treatment with pulsed intravenous methylprednisolone (3x 500 mg), plasma exchange (4-litre exchanges), and intravenous immunoglobulin (400 mg/kg). Serum creatinine remained significantly raised at 255 *μ*mol/L (Figure 1(b)).

As a possible cause for TMA, tacrolimus was stopped. Immunosuppression then comprised mycophenolate mofetil 500 mg twice daily and prednisolone 5 mg daily.

The biopsy was further examined, and, in light of the extent of the glomerular thrombotic process and endothelial hypercellularity, a native immunoperoxidase panel was performed. This revealed a “full house” immunoprofile (IgA++, IgG++, IgM++, C3++, and C1q++) within the glomeruli, with deposition in the mesangial matrix and also at subendothelial loci (Figure 2), consistent with an immune complex mediated glomerulonephritis. This finding triggered both virological and lupus screening.

Initial HBV testing revealed the following: HBsAg detected; hepatitis B e antigen detected; hepatitis B e antibody not detected; hepatitis B core IgM not detected; HBV viral load (Roche) HBV DNA log value 8.15. Serology was negative for the following: CMV, BK virus, Epstein-Barr virus, HIV, hepatitis C virus, delta virus, and parvovirus. Lupus and antiphospholipid screening tests were negative, as was* E. coli* 0157 serology. All previous tests for HBsAg had been negative, most recently at the time of transplantation.

Liver function tests, including markers of synthetic function, were normal. He commenced treatment with Entecavir and completed ten plasma exchanges for ongoing TMA with thrombocytopenia.

The source of his HBV infection was sought. He denied any risk factors. His immediate contacts all screened negative for HBV infection. The allograft donor was HBsAg and cAb negative. Hepatitis B genotyping showed a vaccine escape mutant. One possibility is that the HBV cAb result at the time of starting dialysis was a false negative (commercial assays for the detection of HBV cAb can show marked variability in detecting cAb in comparative studies). He could thus have had resolved HBV infection prior to transplantation which later reactivated, with HBV sAb driving viral mutation. Another possibility is new acquisition of mutated HBV, against which the vaccine-induced sAb was not protective. Revisiting his history revealed that he had travelled to Mecca in the months prior to his presentation with AKI; it is possible that HBV could have been acquired during ritual head-shaving.

A repeat allograft biopsy was performed after two weeks of Entecavir treatment. This showed resolution of the TMA, but with residual immune complex mediated glomerulonephritis.

After one month of Entecavir treatment the HBV viral load had dropped from a log value of 8.15 to a log value of 4.65; the creatinine had fallen from 250 *μ*mol/L to 211 *μ*mol/L; the haemoglobin had risen, and the platelet count had normalised. Tacrolimus was not restarted. Two weeks later, both the HBV viral load and serum creatinine had further improved to 24884 IU/mL and 171 *μ*mol/L, respectively. Several months later, transplant function remains stable at this new baseline and HBV viral load continues to fall.

We hypothesise that the acute allograft dysfunction was secondary to an infection associated (HBV) immune complex mediated glomerulonephritis. Our patient developed acute HBV despite successful HBV vaccination before transplant.

## 3. Discussion 

It is widely accepted that patients receiving RRT should be vaccinated against HBV, ideally well in advance of ESKD. Anti-HBs levels should be checked annually and boosted as indicated [2, 6, 7].

The importance of preventing HBV infection among solid organ transplant candidates and recipients is twofold; immunosuppressed individuals are more susceptible to infection with HBV and if infected with HBV are more likely to develop chronic HBV infection. Furthermore, a transplant candidate may be offered an organ from a HBsAg-negative, HBcAb-positive (“core-positive”) donor, so there is additional benefit to completing HBV immunisation before transplantation [8].

If a kidney transplant recipient has not been vaccinated against HBV prior to transplantation then current best practice is to vaccinate between 2 and 6 months after transplant, thereby avoiding the risk that high dose immunosuppression may blunt the vaccine response [9]. The response to HBV vaccine administration after transplantation varies greatly, with response rates ranging between 17 and 89% [10–12]. Unfortunately, HBsAb levels decline more rapidly in immunosuppressed patients, even in those who develop protective levels [10, 13], and booster doses produce blunted responses [10]. These results underscore the value of pretransplant immunisation, ideally prior to the onset of advanced kidney failure.

The need for hepatitis B vaccination boosters is controversial and practice varies from country to country. There have however been reports of clinically significant HBV infection in previously immunised dialysis patients in whom production of HBsAb was no longer measurable [1].

It is widely accepted that annual measurement of HBsAb levels inform the use of booster doses of hepatitis B vaccine for dialysis patients. The European Consensus Group on hepatitis B immunity have expanded this recommendation to include patients with impaired immune function [14].

KDIGO [3] and the UK Renal Association [4] recommend HBV vaccination for kidney transplant recipients ideally before transplant, with an antibody check 6–12 weeks after the vaccination course. Annual antibody level checks before and after transplantation are recommended, with a booster to be given if the antibody level drops below 10 mIU/mL.

Our case demonstrates that HBV can cause clinically significant disease after transplantation, even when a patient has been vaccinated against HBV before transplantation with apparently protective response. This case is an illustrative example of the possible benefits of following the current International and UK Renal Association guidance.

This case is also notable for the association between acute HBV infection and biopsy-proven TMA with immune complex mediated glomerulonephritis causing acute allograft dysfunction. HBV infection is associated with a range of kidney diseases, most commonly membranous nephropathy and mesangiocapillary glomerulonephritis. However, an association or even causative role for HBV in TMA and immune complex mediated glomerulonephritis has not previously been reported.
